# Plant-Based Food: From Nutritional Value to Health Benefits: 2nd Edition

**DOI:** 10.3390/foods14234138

**Published:** 2025-12-02

**Authors:** Qianqian Zhu, Yongqi Yin

**Affiliations:** College of Food Science and Engineering, Yangzhou University, Yangzhou 225009, China

Plant-based foods are made from plant seeds, fruits, or tissue components that provide direct or processed energy and nutrients for human consumption. They primarily include grains, tubers, legumes and their products, vegetables, and fruits. These plant-based foods not only provide essential nutrients like proteins, vitamins, and minerals, as well as energy, but they also contain a large number of bioactive compounds like terpenoids, polyphenols, saponins, polysaccharides, and flavonoids, which have a variety of physiological functions such as antioxidant, anti-inflammatory, antimicrobial, and antitumor properties [1,2,3]. Consumer and scientific interest in plant-based foods is at an all-time high, owing primarily to climate change mitigation (plant-based diets can reduce greenhouse gas emissions by 29–70% when compared to animal-based diets [4,5]), chronic disease management (such as obesity, type II diabetes, and hypertension) [6,7,8], sustainable environmental stewardship, and efficient food resource utilization [9]. According to the United Nations 2030 Sustainable Development Goals, plant-based foods will play an important role in future food systems, nutritional provision, and public health [10]. Simultaneously, this momentum has pushed researchers to continue deepening their understanding of plant-based foods, including the investigation and development of germplasm resources from various plant species, the impact of environmental conditions and processing technologies on the nutrition and quality of plant-based foods, and the molecular mechanisms by which bioactive substances in plants exert targeted health effects [11].

Based on these critical problems, this special issue “Plant-Based Food: From Nutritional Value to Health Benefits: 2nd Edition” focuses on interdisciplinary research (food science, agronomy, nutrition, and clinical medicine) on plant-based foods. This special issue brings together ten original research articles and one review article from 13 research teams in nine countries, giving essential insights for plant-based food production and tackling health concerns through high-quality publishing.

Comprehensive investigation of plant resources in nature to uncover new sources of nutrition, functionality, and natural components lays the groundwork for the plant-based food industry’s long-term, environmentally friendly development [12]. The possible health advantages and nutritional qualities of *Syzygium paniculatum* Gaertn (a berry extensively grown in Ecuador) remain unknown.

Carrera-Cevallos et al. (Contribution 1) conducted a thorough evaluation of this berry’s morphology, nutritional value, chemical composition, structural properties, and antioxidant capacity at two maturation stages. Their findings showed that the size and weight parameters of this fruit are equivalent to commercial berries such as blueberries, with a high moisture content (88.9%), dietary fiber (3.56%), and protein (0.63%), while being low in fat. The plant has a variety of phenolic compounds, including gallic acid, chlorogenic acid, hydroxycinnamic acid, and ferulic acid, with consumer maturity stage berries being high in anthocyanins and flavonoids, indicating stronger antioxidant activity and free radical scavenging potential. This study found that *Syzygium paniculatum* Gaertn berries are nutritious, low-calorie food candidates with potential uses in functional meals, medicinal formulations, and food preservation. Their findings are a useful resource for pushing for more inquiry and sustainable use of indigenous berry species in functional food systems. Vrca et al. (Contribution 2) investigated the chemical properties, in vitro digestion, and bioactivity of Kumquat (*Citrus japonica* var. margarita) essential oils extracted using two techniques. Their findings indicated that the dominant constituent in both kumquat essential oils was limonene (>93%), which remained stable (>90%) before and after in vitro digestion. Furthermore, kumquat essential oils revealed strong antioxidant activity. This work positions kumquat essential oil as a multifunctional plant component for food preservation (antimicrobial) and nutritional supplements (antioxidant, anticancer), highlighting the value of citrus secondary metabolites beyond flavor enhancement.

*Perilla frutescens* (L.) Britt, as a versatile herbaceous plant widely distributed across Asia, has long been utilized in traditional medicine and culinary practices in China, Japan, Korea, and other Asian countries [13]. Yi et al. (Contribution 3) comprehensively reviewed the chemical composition, pharmacological mechanisms, and industrial applications of *P. frutescens* in food and health products. They summarized the chemotypes and bioactive substance composition and characteristics in different tissue parts of *P. frutescens*, and introduced current applications of *P. frutescens* in functional foods, pharmaceuticals, and cosmetics industries. Additionally, they compiled advances in the application of new biological and genetic improvement methods such as genome editing technologies (CRISPR-Cas9) for enhancing *P. frutescens* yield and bioactive content. This study presents a systematic research framework for the comprehensive development and utilization of perilla, proposing scientific development pathways through the integration of resource development, mechanism research, technology transfer, and standardization, thereby contributing to the optimization of perilla’s multifunctional value and promoting the green transformation of the food industry.

The nutritional quality and functional potential of plant-based foods are inextricably linked to their growth conditions and processing processes. Many plant-based crops (such as *Chenopodium quinoa*, *Trachyspermum ammi*, and *Cicer arietinum*) grow in marginal conditions (saline soils, dry areas) that are unsuitable for conventional staples, paving the way for food security in the face of climate change [14,15]. However, fully achieving the promise of these crops necessitates a thorough understanding of how external variables (ranging from soil composition and water availability to post-harvest processing) influence their nutrition and bioactivity. Drought stress, for example, can increase phenolic accumulation in plants as a defense mechanism, whereas severe stress can reduce yield and macronutrient quality. Similarly, magnetic field pretreatment (as described in Contribution 4) can accelerate germination and nutritional transformation, but its synergistic effects with abiotic stress (such as NaCl) have not previously been described. Four articles in this special issue look at external circumstances and processing treatments as instruments for boosting plant functional components, offering insights into long-term strategies for improving plant-based food quality. Wang et al. (Contribution 4) studied how NaCl treatment affected growth development and phenolic production in germinated quinoa that had been prepared with a magnetic field. In magnetic field-pretreated germinated quinoa, high NaCl concentrations (≥200 mM) inhibited growth and macromolecule hydrolysis (starch, protein). However, low to moderate NaCl concentrations (50–100 mM) significantly promoted phenolic and flavonoid biosynthesis and enhanced antioxidant systems. This improvement was achieved by increased levels of critical enzyme activities and matching genes in the phenylpropanoid pathway, as well as improved antioxidant capacity. Notably, the combined impact of 100 mM NaCl and magnetic field pretreatment was the largest, suggesting that controlled stress may be used to improve quinoa’s functional value, an approach that is also relevant to the functional food development of other pseudocereals (such as buckwheat and sorghum). Xue et al. (Contribution 5) compared changes in growth development, bioactive substance content, and nutritional composition during germination of two chickpea varieties. They found that compared to ungerminated seeds, total flavonoid content in germinated chickpeas of both varieties (Xinying No.1 and Xinying No.2) increased by 3.95-fold and 3.25-fold respectively, while total phenolic content increased by 2.47-fold and 2.38-fold. Germination also significantly increased soluble protein, free amino acid, and total dietary fiber content in chickpea sprouts while reducing resistant starch and insoluble dietary fiber content, enhancing chickpea bioavailability.

In recent years, nanofertilizer application has improved productivity, reduced production costs, minimized the impact of biotic and abiotic stress on crop growth, and promoted agricultural transformation [16,17]. Sobatinasab et al. (Contribution 6) first evaluated changes in essential oil content, chemical composition, flavonoid content, phenolic content, and antioxidant capacity of ten different Ajowan (*Trachyspermum ammi*) populations under different amounts of nano-silicon (0, 1.5, and 3 mM) and three irrigation regimes (50%, 70%, and 90% field capacity). Results showed that thymol, carvacrol, p-cymene, and γ-terpinene were identified as major oil components. Under 50% field capacity (severe drought), nano-silicon (1.5–3 mM) restored Ajowan essential oil yield and regulated its chemical characteristics: in the Khorbir population, the major antimicrobial component thymol reached 65.71% (50% irrigation, no nano-silicon), while nano-silicon supplementation enhanced phenolic and flavonoid compound accumulation (e.g., 46.78 mg Quercetin/g Dry Weight in Ardabil population) and antioxidant activity. This work emphasizes nano-silicon’s role in maintaining plant cell integrity under water stress while enhancing bioactive metabolites—a promising green technology for plant-based food production in arid regions. Seephua et al. (Contribution 7) used watermeal (*Wolffia globosa*) in noodle manufacturing and investigated its impacts on noodle physicochemical qualities, antioxidant activity, and starch and protein digestion. Their findings revealed that adding watermeal considerably improved noodle quality, boosted protein and chlorophyll content, and raised bioactive chemical and antioxidant activity in noodles. In addition, the amount of resistant and slowly digesting starch in noodles rose, considerably boosting protein digestibility. This study validated watermeal’s promise as a sustainable plant-based ingredient for making nutritious noodles.

The special issue’s central focus is the translation of plant-based dietary components into evidence-based health benefits. Alcohol use is a global public health concern, with hangover symptoms (such as gastrointestinal pain and headaches) impacting 70–80% of drinkers, and persistent alcohol intake causing liver damage [18,19]. Traditional plant remedies have long been utilized to ease these symptoms, but scientific research on their effectiveness and safety is lacking [20]. Two randomized, double-blind, placebo-controlled clinical trials included in this special issue assessed the efficacy of Hovenia dulcis extract, which has long been used in East Asian medicine to treat alcohol intoxication, in reducing hangover symptoms. Lee et al. (Contribution 8) and Paik et al. (Contribution 9) found that mixing *Hovenia dulcis* extract with *Pueraria lobata* extract (HDPB) or glutathione-rich yeast effectively lowered blood alcohol and acetaldehyde levels in healthy people aged 19 to 40. Specifically, as compared to placebo, HDPB lowered blood alcohol levels by 7–10% at 0.5–6 h after drinking and reduced gastrointestinal problems (a prominent hangover symptom) by 40–50%. These extracts increased alcohol dehydrogenase and aldehyde dehydrogenase activity while decreasing oxidative stress, giving clinical evidence that *H. dulcis*-based formulations are safe plant-based hangover treatments. These findings integrate ancient knowledge with current clinical research, laying the groundwork for functional beverage development aimed at alcohol-related applications.

Foodborne pathogens (such as *Escherichia coli* and *Staphylococcus aureus*) and oxidative stress pose significant risks to food safety and human health. Plant essential oils (EOs) have emerged as potential natural alternatives to synthetic preservatives and antioxidants, but their bioavailability and selectivity (e.g., attacking pathogens while sparing beneficial microorganisms) are crucial concerns [21]. Vrca et al. (Contribution 2) discovered that kumquat essential oil had broad bioactivity, inhibiting E. coli and S. aureus growth, reducing *E. coli* adhesion to polystyrene surfaces (>1 log), and demonstrating cytotoxic activity against cervical cancer cell lines (HeLa), human cancer cell lines (HCT116), and human osteosarcoma cell lines (U2OS) with IC50 values of 1–2 mg/mL while causing minimal harm to healthy cell lines (RPE1).

Sarcopenia, or gradual loss of muscle mass and function, affects 10–20% of persons over the age of 65, increasing the risk of falls, disability, and death [22]. Current therapies (such as physical exercise and protein supplements) are beneficial but frequently underused, emphasizing the need for dietary measures. In this special issue, Na et al. (Contribution 10) undertook a large-scale prospective analysis (28,229 people, 9.37-year follow-up, UK Biobank) to investigate the correlations between dietary nitrate (53.95%) and water (37.89%) and sarcopenia risk. Low grip strength, poor walking speed, and sarcopenia/pre-sarcopenia were all adversely correlated with increased nitrate consumption, according to adjusted logistic regression models. Stratified analysis further revealed gender- and age-specific effects: women benefited from increased grip strength and reduced Sarc-Presarc risk, while men showed improved walking speed; adults over 65 demonstrated stronger associations between nitrate and preserved muscle mass. Mediation analysis identified protein homeostasis (serum albumin, creatinine) and blood pressure as key pathways. This study provides population-level evidence identifying dietary nitrate (primarily from vegetables) as a novel protective factor, with higher intake associated with reduced sarcopenia/pre-sarcopenia risk and maintained muscle function, offering a low-cost, scalable strategy for sarcopenia prevention in aging societies.

Ahn et al. (Contribution 11) screened compounds with anti-photoaging properties from *Kaempferia parviflora*, successfully isolating five methoxyflavones. Research showed that all methoxyflavones effectively inhibited tumor necrosis factor-alpha-induced reactive oxygen species production. Some compounds significantly reduced matrix metalloproteinase-1 levels in normal human dermal fibroblasts but did not induce significant increases in collagen secretion. These compounds inhibited Extracellular signal-regulated kinase phosphorylation in a concentration-dependent manner, thereby regulating mitogen-activated protein kinase pathways. This study provides new candidate compounds for developing natural anti-skin aging products and validates their protective effects and potential mechanisms. Yi et al. (Contribution 3) found that volatile oils, flavonoids, and phenolic acid compounds in *P. frutescens* exhibited considerable pharmacological activity in a variety of experimental paradigms. Perillaldehyde, for example, has strong antibacterial and anti-inflammatory characteristics, limonene is anticancer, antioxidant, and antiviral, and rosmarinic acid is anti-inflammatory and antioxidant. They stated that while perillaldehyde and perillyl alcohol are typically considered harmless, the potential toxicity of perillaldehyde, notably hepatotoxicity and neurotoxicity at large dosages, warrants more investigation. Future research should therefore focus on comprehensive safety assessments and clinical studies of perilla bioactive compounds, utilizing advanced analytical techniques to further elucidate pharmacological mechanisms and metabolic pathways of their complex chemical structures in order to support high-value applications in pharmaceuticals, functional foods, and the sustainable industries.

This special issue’s cutting-edge research increases our understanding of plant-based diets from “farmland to health.” Controlled stress (NaCl, drought) and exogenous stimuli (magnetic fields, nano-silicon) can alter plant metabolites (phenolics, essential oils) to improve nutrition. Plant extracts and secondary metabolites (kumquat essential oils) address specific requirements (hangovers, infections, and cancer), and clinical and in vitro evidence supports their safety and efficacy. Large cohort data link plant nutrients (nitrates) to lower sarcopenia risk, highlighting the importance of plant-based diets in aging-related health. However, existing limitations in plant-based food research remain, with most studies focused on single plant species or components; future study should look into synergistic effects across plant-based diets. Furthermore, long-term clinical studies are uncommon, particularly for plant therapies targeting chronic illnesses, and mechanistic understanding of how plant components (such as limonene and nitrates) interact with human microbiomes or immune systems is lacking. Moving forward, merging metabolomics, microbiomics, and customized nutrition will be critical development paths for realizing the full potential of plant-based diets. Plant-based foods are considerably more than just animal-free alternatives or niche dietary options; they are critical components in tackling global concerns such as climate change and chronic illnesses, as well as rich sources of tailored nutrients and bioactive chemicals. Interdisciplinary collaboration between agronomists maximizing crop development, food scientists improving bioavailability, nutritionists establishing tailored diets, and physicians evaluating health consequences is critical for realizing the full potential of plant-based meals. We anticipate that this special issue will stimulate more plant-based food innovation and discussion, advancing our goal of a nutrient-dense, sustainably produced food system.

## Data Availability

Not applicable.
